# Macrofungal Mediated Biosynthesis of Silver Nanoparticles and Evaluation of Its Antibacterial and Wound-Healing Efficacy

**DOI:** 10.3390/ijms25020861

**Published:** 2024-01-10

**Authors:** Gayathri Vijayakumar, Hyung Joo Kim, Jeong Wook Jo, Senthil Kumaran Rangarajulu

**Affiliations:** 1 Department of Biotechnology, Rajalakshmi Engineering College, Chennai 602105, India; gayathri.vijayakumar@rajalakshmi.edu.in; 2 Department of Biological Engineering, Konkuk University, Seoul 05029, Republic of Korea; hyungkim@konkuk.ac.kr (H.J.K.); jjw9802@naver.com (J.W.J.)

**Keywords:** antimicrobial activity, silver nanoparticles, macrofungi, mycobiosynthesis, wound-healing activity

## Abstract

Recently, the utilization of biological agents in the green synthesis of nanoparticles has been given interest. In this study, silver nanoparticles were synthesized from an aqueous extract of macrofungus (mushroom), namely *Phellinus adamantinus*, in a dark room using 20 µL of silver nitrate. Biosynthesized silver nanoparticles were confirmed by analyzing them using a UV-Vis (ultraviolet-visible) spectrophotometer. The synthesized silver nanoparticles were optimized at different pH and temperatures with various dosages of AgNO_3_ (silver nitrate) and fungal extracts. The synthesized AgNPs (silver nanoparticles) were characterized using TEM (transmission electron microscopy) and EDX (energy-dispersive X-ray) analyses, which confirmed the presence of silver nanoparticles. The size of the nanosilver particles was found to be 50 nm with higher stability. The mycosynthesized AgNPs showed effective antibacterial activity against strains of Gram-positive (*Staphylococcus aureus* and *Bacillus subtilis*) and Gram-negative (*E. coli* and *Pseudomonas aeruginosa*) bacteria. The minimum inhibitory concentration (MIC) was found to be 3.125 μg/mL by MIC assay. The MTT assay (3-[4,5-dimethylthiazol-2-yl] 2,5-diphenyl-2H-tetrazolium bromide) was performed to study cytotoxicity, and reduced cell viability was recorded at 100 μg/mL. Silver-Polygalacturonic acid-Polyvinyl alcohol ((Ag-PGA)-PVA) nanofiber was prepared using the electrospinning method. The in vitro wound scratch assay was demonstrated to study the wound-healing efficacy of the prepared nanofiber. The wound-healing efficacy of the AgNP-incorporated nanofiber was found to be 20% after 24 h. This study will lay a platform to establish a unique route to the development of a novel nanobiomaterial and its application in antibacterial and wound-healing therapy.

## 1. Introduction

Over the past few years, one of the sciences that has developed the fastest is nanotechnology. This multidisciplinary field of study integrates concepts from engineering, physics, chemistry, biology, and material science [1,2,3]. The study, creation, and application of materials in the physical range of 1–100 nm is known as nanotechnology. The fundamental building block of nanotechnology is thought to be nanoparticles [4,5]. Nanoparticles reveal atom-like behavior due to their high surface-to-volume ratio and the wide gap between the valence band and the conduction band [6,7]. One of the important aspects in the field of nanotechnology is the development of a more consistent process for the synthesis of nanomaterials with variable size ranges with good monodispersity, shape, chemical composition, and their potential use for human benefit. Metal nanoparticles possess exceptional physiochemical features, such as optical, catalytic, electrical, magnetic, and antibacterial qualities, which contribute to their high specific surface area and surface atoms [8]. Nanoparticles are a secure, practical, and affordable way to tackle future difficulties [9].

Silver is a safe and efficient bactericidal metal that has been employed among other metals. It is non-toxic to animal cells but toxic to bacterial cells [10,11]. Ever since ancient times, wounds, burns, and various bacterial illnesses have been treated with metallic silver and silver sulfadiazine, known as silver [12]. Applications for silver are many and include the biomedical industry, food production, water and air purification, cosmetics, apparel, and many domestic products [13]. Silver nanoparticles are the most important candidates for solving various medical problems due to their chemical biocompatibility, inertness, oxidation resistance, and very little chance of generating drug-resistant microbial strains [14]. Silver nanoparticles have a wide spectrum of antimicrobial activity against a diverse range of bacteria by reducing the fitness of the bacteria in biofilms [7,15,16]. Hence, they are used to treat infectious diseases caused by human pathogens and eliminate multidrug resistance problems [9]. Silver nanoparticles have been demonstrated to have inherent cytotoxic action and may decrease both the quantity of pathogens in a wound and the inflammatory response [1,17]. They have a broad biocidal impact on microorganisms by rupturing their unicellular membrane, which disrupts their enzymatic activity and ultimately causes the cells to die [18]. AgNPs exhibit a multitude of bioactivities, including cytotoxicity, antimicrobial, antiviral, antifungal, and anticancer properties [14]. Since it has antiviral activity, it is used against several viral diseases, including hepatitis B, respiratory syncytial virus, herpes simplex virus type, and monkeypox virus [17]. It is utilized in skin ointments, topical creams to prevent burns and open wounds from becoming infected, wound dressings, antiseptic sprays, and antiseptic materials [8,18]. As a result, AgNPs have significantly impacted a broad range of applications across several industries, such as the food, textile, chemical, and agricultural industries, medication delivery, ointments, nanomedicine, chemical sensing, data storage, and cell biology [19,20]. Antimicrobial and antioxidant properties have also been applied to them [21].

The three main methods for the synthesis of nanoparticles are physical, chemical, and biological methods. Both physical and chemical methods require high temperatures, huge amounts of toxic chemicals and hazardous reagents, and high amounts of energy and are costly [22]. These disadvantages in physical and chemical methods led to increasing interest in biogenic synthesis methods. The biological method involves the utilization of plants, enzymes, and microbes such as bacteria, yeast, and fungi [23] to synthesize reliable, clean, simple, sustainable, cost-effective, and protein-capped nanoparticles with good dispersity. Among the biosystems, plants and microbes are specially considered as a potential source of biofunctional natural products, which is exploited by using them as reducing agents in the synthesis of different nanomaterials [24,25,26,27].

Recently, the application of fungal materials in nanosynthesis has been given attention in the field of myco-nanobiotechnology [4,28]. Among these, fungal mushrooms (macrofungi) are the source of a wide range of bioactive metabolites (viz. terpenoids, phenols, polysaccharides, and lectins) [29,30]. They yield a lot of extracellular enzymes and protein metabolites, and they have a high nutritional value. These enzymes reduce the toxicity of the material by acting as a bio-reducing agent and stabilizing agent during the creation of nanoparticles [31]. Fungi that resemble mushrooms are easy to work with and cultivate and have strong wall-binding and intracellular metal absorption capacities. They can also be used to quickly create vast amounts of nanoparticles [4]. They are easier to grow at both laboratory and industrial levels, and the yield is high [9,32]. They are inexpensive substrates and produce a wide range of commercially interesting metabolites [33]. Antioxidant, anti-inflammatory, antitumor, antiviral, antibacterial, anticancer, and other properties are present in mushrooms. [34]. The use of UV-Vis spectroscopy has allowed researchers to examine the kinetics of the fungal-derived nanoparticle process. Additional analysis has revealed unique optical, physical, and chemical characteristics, including high quantum yield, outstanding biocompatibility, and high photostability [31,33]. Thus, mushrooms have attracted considerable interest in the field of nanotechnology [35]. Though several reports on the biogenic synthesis of nanosilver are available [36,37,38], its application in cytotoxicity studies should be elaborated for the development of potential antibacterial and wound-healing agents. It has been reported that medicinal mushrooms such as *Phellinus* spp., *Volvariella* sp., *Inonatus* sp., and *Schizophyllum* sp. act as bioreductant candidates in the synthesis of nanoparticles [34,36,39].

The macrofungus *Phellinus adamantinus* is an important medicinal mushroom and considered a reservoir for a wide range of phytochemicals (viz. alkaloids, saponins, glycosides, flavonoids, tannins and phenolic compounds, resins, steroids and sterols, thiols, polysaccharides, and proteins) [40]. Though the fungus is reported to show antioxidant, antitumor, antimicrobial, and free radical scavenging potentials, its bioactivity must be tested for its potential in the synthesis of nanosilver [40]. Further investigation on screening the fungal extract is essential to analyze the reducing property involved in the production of silver NPs and also to prolong their extended stability. The mycosynthesized nanoparticles were also tested for their antimicrobial activity against a range of human pathogenic microbes. This study will demonstrate an encouraging outcome in terms of cytotoxicity [16]. Hence, in this investigation, extracts of *Phellinus adamantinus* were used to synthesize AgNPs. In addition, nanosilver was evaluated for its potential antibacterial, cytotoxicidal, and wound-healing activities.

## 2. Results and Discussion

### 2.1. Nanoparticle Synthesis

**Figure 1:**
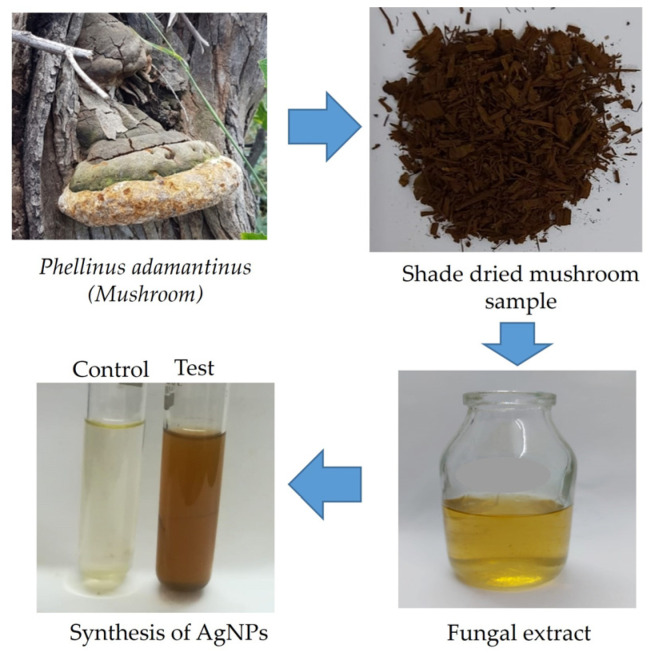
Process involved in the biosynthesis of nanosilver from *Phellinus adamantinus* extract treated with silver nitrate solution and incubated at 37 °C in a dark static environment; it turned dark brown after 24 h.

Synthesis of silver nanoparticles was performed by adding 20 μL of 1 mM AgNO_3_ to mushroom extracts (*Schizophyllum commune*, *Volvariella volvacea*, *Phellinus adamantinus*, and *Inonatus porrectus*) after being combined in a 1:9 ratio each with distilled water. The change of color from colorless to reddish brown confirmed that silver nanoparticles were synthesized from mushroom extracts. Of all the four mushroom extracts, *Phellinus adamantinus* maintained a reddish-brown color and showed good stability in a dark environment at room temperature. The current investigation was supported by the earlier researcher’s documentation [29] that silver nanoparticles synthesized from *E. scabrosa* mushroom extract demonstrated good stability [41]. These results reveal that the silver nanoparticles synthesized from mushroom extracts have good stability (Figure 1). The synthesized silver nanoparticles were characterized by UV-Visible spectroscopy, TEM analysis, and EDX analysis. The antimicrobial effect was tested for mushroom extract and the synthesized silver nanoparticles.

### 2.2. UV-Vis Spectrum Analysis

**Figure 2:**
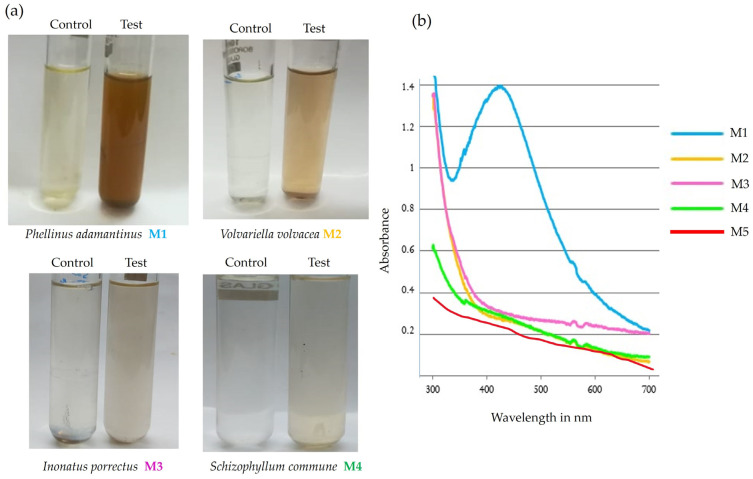
(**a**) The mushroom extracts of 4 fungal species treated with silver nitrate solution showing a color change indicating the synthesis of AgNPs. (**b**) UV-Vis spectra of AgNPs synthesized from different mushroom samples—(M1) *Phellinus adamantinus*, (M2) *Volvariella volvaceae*, (M3) *Inonatus porrectus*, (M4) *Schizophyllum commune*, and (M5) fungal extract of *Phellinus adamantinus* without silver nitrate (negative control).

The mycosynthesized silver nanoparticles were analyzed by UV-Visible spectroscopy for the presence and stability of silver nanoparticles in the range of 300 nm to 700 nm. The excitation of surface plasmon resonance (SPR) in silver nanoparticles is the reason for the formation of the reddish-brown color, and it confirmed the presence of silver nanoparticles [42]. The sharp peak was obtained at 423 nm by UV-Vis spectroscopy for the silver nitrate solution with *Phellinus adamantinus* (M1) after 24 h of incubation. The extract of *Mushroom* without AgNPs (control) does not show any peak in the range of 300 nm to 700 nm (Figure 2) [43,44].

### 2.3. Stability

**Figure 3:**
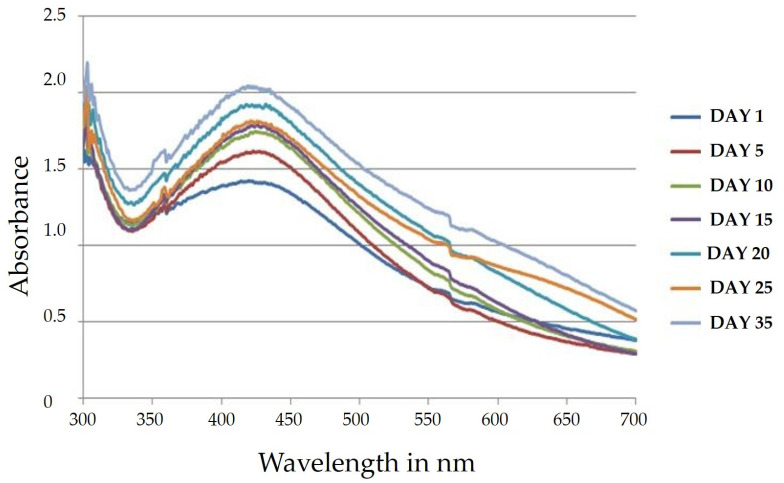
Comparison of the stability of synthesized silver nanoparticles on the 1st day, 5th day, 10th day, 15th day, 20th day, 25th day, and 35th day using *Phellinus adamantinus* extract.

The stability of the mycosynthesized silver nanoparticles was recorded using UV-Visible spectroscopy at different time durations. It is evidenced that with respect to the increase in time of incubation, the peak becomes sharper [45]. After 24 h of incubation, SPR showed a peak at 423 nm. After 48 h, the peak switched to 418 nm. The 30-day-old sample displayed a stable peak at 418 nm (Figure 3). This proves that the synthesized AgNPs were stable after 48 h. The spectrum peak was found to be 418 nm, even on the 30th day, which proved that the synthesized AgNPs maintained stability. This denoted the stability of silver nanoparticles synthesized from *Phellinus adamantinus* extract [44].

### 2.4. TEM Analysis

**Figure 4:**
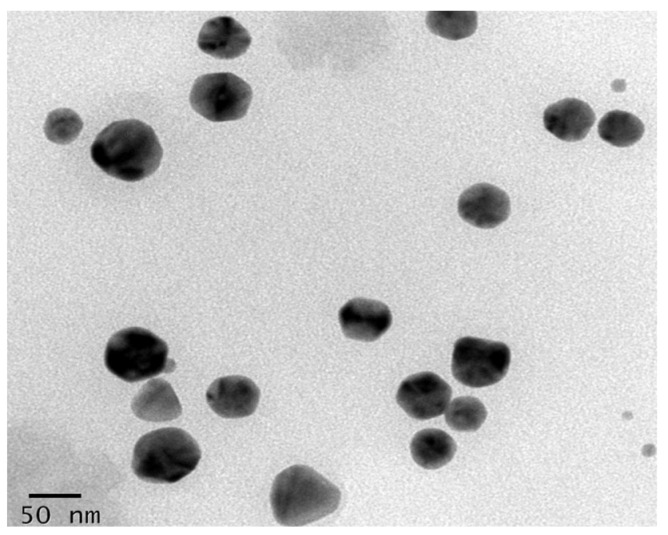
Transmission electron microscopic examination of *Phellinus adamantinus* extract-synthesized silver nanoparticles shows the nano size of 50 nm with sphere-shaped AgNPs synthesized.

The optimized silver nanoparticles (AgNPs) synthesized from *Phellinus adamantinus* were characterized for their morphology and size. The TEM images of mycosynthesized silver nanoparticles at 50 nm are shown in Figure 4. The particles are spherical in shape with a size range of 50 nm. In previous studies, spherical and hexagonal particles 22–30 nm in size were mycosynthesized with the use of *Fusarium oxysporum* cultural liquid [46]. In other studies, the silver nanoparticles synthesized from the leaf extract of *Cassia roxburghii* were spherical in shape with a diameter range from 10 to 30 nm [47], and silver nanoparticles from *Acacia leucophloea* extract were reported to be spherical in shape with a diameter of 17 to 29 nm [48]. Silver nanoparticles from another mushroom, namely *Flammulina velutipes* extract, were stated to range from 8 to 33 nm [49], which was similar to that of the present study.

### 2.5. EDX Analysis

**Figure 5:**
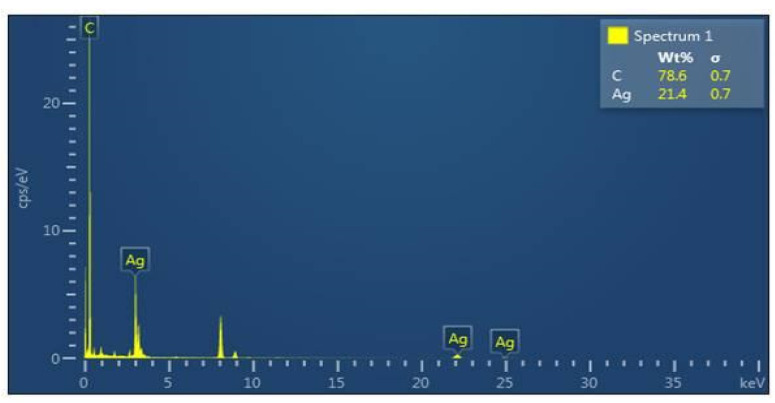
Energy-dispersive X-ray (EDX) spectrum of synthesized nanoparticles confirms the presence of 21.4% silver (Ag), showing absorption peaks at 3 keV.

EDX revealed signals in the silver region and confirmed the presence of silver nanoparticles. The peak at 3 keV confirmed the metal nanoparticles due to surface plasmon resonance (SPR), which is seen in Figure 5. The signal showed that the mycosynthesized silver nanoparticles were free from impurities. Previous studies also reported that silver nanoparticles showed absorption peaks at 3 keV [50]. In another study, it was reported that when AgNPs were analyzed using EDX, the strong signal of Ag atoms showed the crystalline property and the presence of oxygen peaks along with the silver signals showed that AgNPs were capped by phytoconstituents through oxygen atoms [47,51].

### 2.6. Optimization of Silver Nanoparticles

#### 2.6.1. Effect of pH

**Figure 6:**
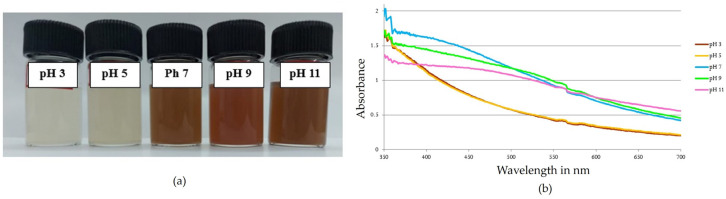
Effect of pH on synthesized AgNPs. (**a**) The AgNP synthesis was observed as a color shift towards brown, indicating that the particle size gradually increases with concentration. (**b**) At pH 7, stable AgNPs were formed, which was confirmed with a spectral peak at 418 nm; the solutions with a pH of 3, 5, 9, and 11 showed unstable peaks.

The mixture, which contained 1 mL of *Phellinus adamantinus*, was extracted with 9 mL of 1 mM AgNO_3_ adjusted to different pH levels (3, 5, 7, 9, 11) and incubated at 37 °C in a dark environment. After 24 h, the formation of a reddish-brown color was observed in pH 7, 9, 11, and no color change was observed in acidic pH, which confirmed the absence of silver nanoparticles. The unstable peaks were observed at basic pH 9 and 11, where a reddish-brown color was observed, which was found to be not stable after 48 h (Figure 6a). In neutral pH 7, the silver nanoparticles were highly stable and showed sharp peaks at 418 nm when analyzed using UV-Vis spectroscopy (Figure 6b). Therefore, the optimum pH was found to be 7 for synthesis of AgNPs. Earlier researchers also documented that pH 7 was the optimum for the synthesis of silver nanoparticles [52,53,54,55].

#### 2.6.2. Effect of Concentration of Silver Nitrate

**Figure 7:**
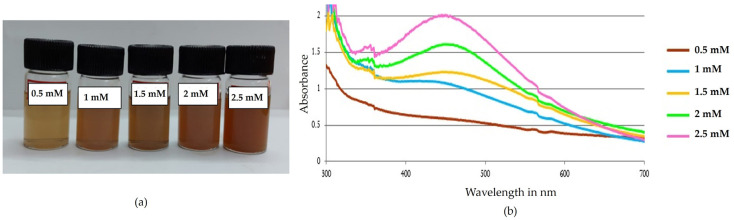
Effect of concentration on synthesized AgNPs. (**a**) The synthesis of AgNPs was observed as a color shift towards red, indicating that the particle size gradually increases with concentration; (**b**) 1 mM concentration of AgNO_3_-synthesized AgNPs, which was confirmed with a spectral peak at 418 nm.

Different concentrations from 0.5 mM to 2.5 mM of silver nitrate were added to the aqueous extract of *Phellinus adamantinus* at pH 7 and incubated for 24 h in a dark environment at 37 °C. There was no color change observed in the 0.5 mM concentration, indicating the absence of silver nanoparticles. In 1 mM to 2.5 mM concentrations of silver nitrate, the formation of a reddish-brown color confirmed the presence of silver nanoparticles (Figure 7a). In the present study, the stability of the synthesized nanoparticles was found to be maintained at a 1 mM concentration of silver nitrate and attained a peak at 418 nm (Figure 7b). In another study, Krishnaraj et al. [54] reported that 1 mM is the optimum concentration for the synthesis of silver nanoparticles from *Acalypha indica* leaf extract.

#### 2.6.3. Effect of Concentrations of Aqueous Extract

**Figure 8:**
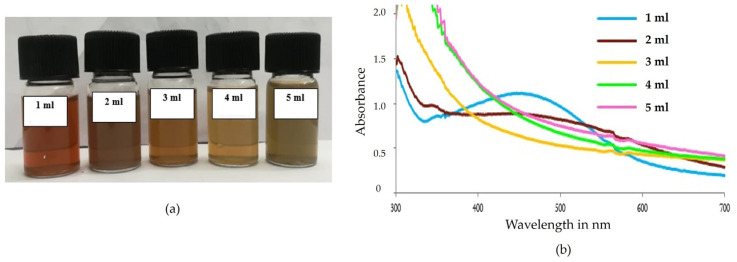
Effect of concentrations of aqueous extract *Phellinus adamantinus*. (**a**) The synthesis of AgNPs was observed as a color shift towards red, indicating that the particle size gradually increases with concentration; (**b**) 1 mL concentration of aqueous extract of *Phellinus adamantinus* revealed stability with a sharp peak at 418 nm of AgNO_3_-synthesized AgNPs, which was confirmed with a spectral peak at 418 nm.

Different volumes of aqueous extracts of *Phellinus adamantinus* were added to 9 mL of 1 mM AgNO_3_ at pH 7 and incubated for 24 h in a dark environment at 37 °C. The formation of a reddish-brown color was observed in all mixtures (Figure 8a). When analyzed using UV-Vis spectroscopy, the spectrum for 1.0 mL showed good stability with a sharp peak at 418 nm (Figure 8b). Other mixtures did not maintain a peak, and sediments were formed [52]. In another study, it was reported that 1 mL was the optimum concentration for the synthesis of silver nanoparticles from *Acalypha indica* leaf extract [55].

#### 2.6.4. Effect of Temperature

**Figure 9:**
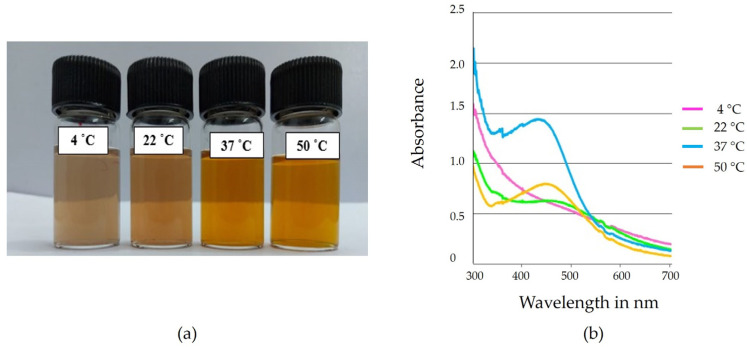
(**a**) Optimization of AgNPs at different temperatures. (**a**) The color shift of AgNP synthesis was found to be towards brown, suggesting a gradual increase in particle size with increasing temperature. (**b**) Stable AgNPs developed at 37 °C, confirming a prominent peak at 418 nm.

A mixture containing 1 mL of aqueous extract and 9 mL of 1 mM AgNO_3_ at pH 7 was incubated at 4 °C, 22 °C, 37 °C, and 50 °C. The latter three temperatures were used to create silver nanoparticles (Figure 9a). AgNPs, however, exhibited a strong peak at 418 nm in their spectrum and were more stable than the others at 37 °C [56] (Figure 9b). Earlier researchers also proved that the reaction temperature could play a key role in particle growth and shape/size control, especially for silver nanoplates [57].

### 2.7. Antibacterial Activity

**Figure 10:**
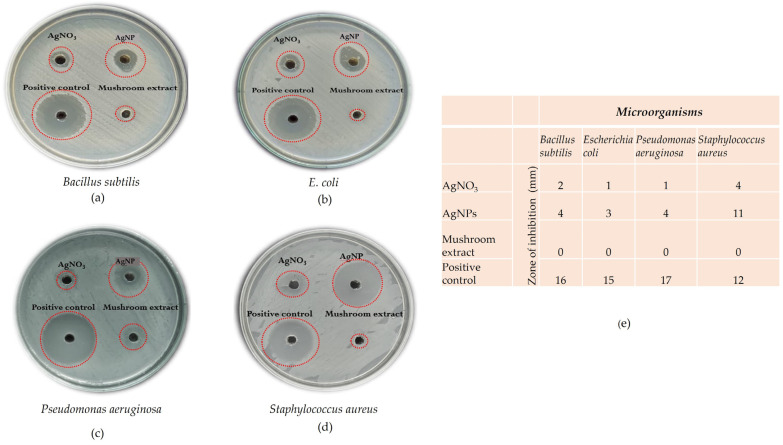
Antibacterial activity of synthesized silver nanoparticles against *Staphylococcus aureus* (**d**) treated with optimized mycosynthesized silver nanoparticles, showing maximum antimicrobial activity when compared to *Bacillus subtilis* (**a**); *Escherichia coli* (**b**); and *Pseudomonas aeruginosa* (**c**). (**e**) The table displays the 10 mm zone of inhibition against *Staphylococcus aureus*, followed by *Bacillus subtilis* (16 mm), *Pseudomonas aeruginosa* (17 mm), and *E. coli* (15 mm).

The antibacterial activity of optimized mycosynthesized silver nanoparticles was tested against both Gram-positive and Gram-negative pathogenic bacteria such as *Bacillus subtilis*, *Staphylococcus aureus* and *Pseudomonas aeruginosa*, *E. coli*, respectively. Among the evaluated four pathogens, *Staphylococcus aureus* was found to be highly sensitive to mycosynthesized silver nanoparticles, with a maximum zone of inhibition (ZOI) of 11 mm, followed by *Bacillus subtilis* and *Pseudomonas aeruginosa* at 4 mm and *E. coli* at 3 mm. The aqueous extract of *Phellinus adamantinus* didn’t show any inhibitory activity against the test pathogens. AgNO_3_ exhibited the least antibacterial activity. The antibiotic streptomycin showed antibacterial activity of 10 mm ZOI towards *Staphylococcus aureus*, followed by 16 mm for *Bacillus subtilis*, 17 mm for *Pseudomonas aeruginosa*, and 15 mm for *E*. *coli* (Figure 10) [58,59].

A number of study teams have previously reported potential mechanisms of cell death, such as DNA losing its capacity to replicate and cellular proteins becoming inactive when exposed to Ag+. Ag+ attaches to the functional groups of proteins, denaturing the protein. Highly reactive metal oxide nanoparticle treatment of bacteria results in a dramatic increase in the permeability of the bacterial membrane, which prevents the bacterial cells from controlling transport across the plasma membrane and ultimately leads to cell death [60]. According to previous studies, the bactericidal effects of AgNPs may be caused by the electrostatic interactions that exist between negatively charged bacterial cells and positively charged AgNPs [61]. Added to that, AgNPs’ physicochemical characteristics have led to changes in them that make them effective antibacterial drugs because of their higher surface area-to-volume ratio [62].

### 2.8. Minimum Inhibitory Concentration (MIC) Analysis of Mycosynthesized AgNPs

**Figure 11:**
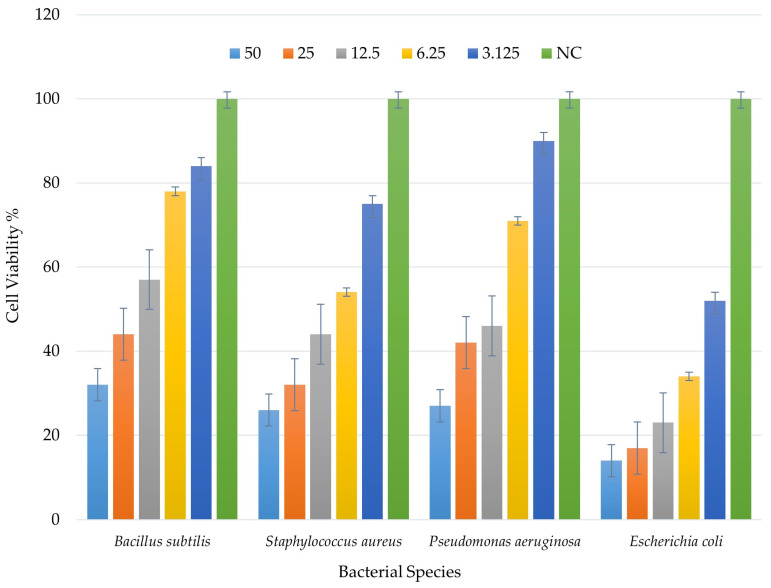
MIC for different bacterial species: *Bacillus subtilis*, *Staphylococcus aureus*, *Pseudomonas aeruginosa*, and *Escherichia coli* treated with *Phellinus*-mediated mycosynthesized AgNP expressed 100% antimicrobial activity at 3.125 µg/mL as its MIC level (NC—negative control).

The lowest concentration of silver nanoparticles at which no growth of pathogenic bacteria was observed is known as minimum inhibitory concentration (MIC). This *Phellinus*-mediated mycosynthesized AgNP came with excellent antimicrobial activity against the test organisms *Bacillus subtilis*, *Staphylococcus aureus*, *Pseudomonas aeruginosa*, and *Escherichia coli*, as shown in Figure 11. The MIC was found to be 3.125 µg/mL for all the test organisms. This study is similar to one of the previous reports [63]. This significant effect of AgNPs may be due to the proteins and other biomolecules of the mushroom extract bound to the surface of AgNPs, as the antimicrobial activity of mushrooms is well established. In another study [64], it was reported that the minimum inhibitory concentration (MIC) was significantly lower than that of antibiotics (positive control), suggesting up to 100 times higher effectiveness of the synthesized AgNPs.

### 2.9. In Vitro Cytotoxicity

**Figure 12:**
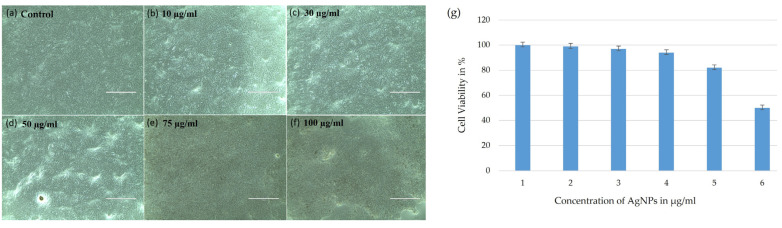
(**a**–**f**) MTT assay of biosynthesized AgNPs against Vero cells at different concentrations of AgNPs: (**a**) control, (**b**) 10 μL, (**c**) 30 μL, (**d**) 50 μL, (**e**) 75 μL, and (**f**) 100 μL. The survivability of the cells decreased (**g**) with an increase in the concentration of synthesized AgNPs. The size measurement of the scale bar line is 500 (**a**–**f**). Experiments were repeated thrice, and (**g**) the graph indicates the percent of cell viability at different dosages of nanosilver treatments. The error bar is presented as mean ± SD, which is statistically significant with *p* value < 0.05.

The MTT (3-(4,5-dimethylthiazol-2-yl)-2,5 diphenyltetrazolium bromide) assay was performed to monitor the inhibitory activity of synthesized AgNPs. The different concentrations (10, 30, 50, 75, and 100 µg/mL) of AgNPs were tested on the Vero cell line under in vitro conditions (Figure 12). Vero cells treated with mycosynthesized AgNPs and untreated Vero cells (control) incubated for 48 h were examined under an inverted microscope. Vero cells treated with AgNPs showed cell shrinkage and altered morphologies, but untreated cells showed no changes in form. AgNPs’ IC50 was measured at a value of 100 µg/mL. Vero cells treated with a greater dosage of 100 µg/mL exhibited approximately a 50% reduction in viability, as shown in Figure 12. AgNP surface characteristics are thought to play a significant role in the absorption of nanomaterials by cells [65]. The penetration of nanoparticles into cells is caused by the surface charges of AgNPs [66]. An earlier study found the IC50 value to be 62.8 µg/mL against Vero cells [67]. In another study, the anticancer effect of AgNPs synthesized from white rot fungi was analyzed, which revealed the relationship between the % of cell viability and % of cell inhibition for A549 cell lines and the concentration of the used nanoparticles. As the concentration of the nanoparticles decreased, the viability of the A549 cell lines dose-dependently reduced [68]. This was found to be proven in the present study, where the cell viability decreased to 50%, whereas the cell viability was found to be less than 50% when the concentration of AgNPs was less than 100 µg/mL.

### 2.10. AgNP-Incorporated Nanofiber

**Figure 13:**
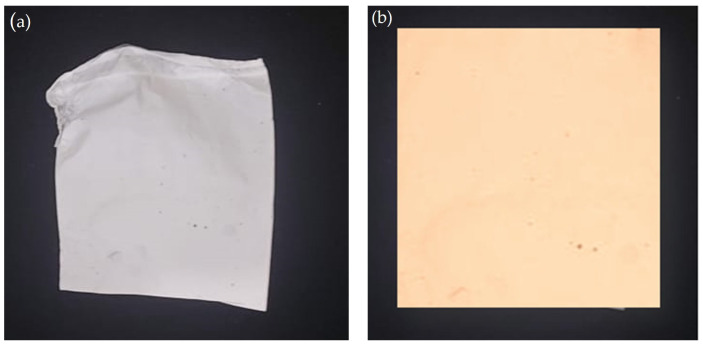
(**a**) PVA nanofiber appears white in color; (**b**) AgNP-incorporated nanofiber appears pale brown, indicating nanofiber with incorporated silver nanoparticles.

Polygalacturonic acid (PGA) in this current study was used as a natural candidate for reducing silver ions into silver nanoparticles; in the meantime, PGA was used as a good stabilizer for the formed AgNPs, too [69]. With a high voltage of 11 kV supplied, the spinnable solution (*(Ag-PGA)-PVA* spinnable solution) was introduced into the electrospinning system at a feed rate of 1.3 mL/h. The spinnable solution was used to generate nanofibers on aluminum foil at room temperature. When they were dried, they were taken out of the aluminum foil and used for more research. In Figure 13a,b, the white color indicates PVA without AgNPs, and pale brown indicates nanofiber with incorporated silver nanoparticles (1% Ag-PGA with 8% PVA).

In contrast to PVA nanofiber without Ag-PGA, which demonstrated no zone of inhibition against *Escherichia coli* and *Staphylococcus aureus*, 1% Ag-PGA combined with 8% PVA demonstrated a larger zone of inhibition. This proves that PVA nanofiber with Ag-PGA expressed an antibacterial effect. In a previous study, in comparison to plain nanofiber, the antibacterial investigations showed a considerably larger zone of inhibition of PVA–chitosan nanofibers loaded with Ag nanoparticles and sulfanilamide against *Staphylococcus aureus*, *Escherichia coli*, and *Pseudomonas aeruginosa* [70]. AgNPs were identified as the cause of the composite nanofibers’ antibacterial activity (Table 1). The explanation that has been suggested is that AgNPs accumulated within the bacterial cell wall, increasing the permeability of cell membranes [71]. According to a different theory, AgNPs interacted with the thiol groups (-SH) of compounds made of cysteine and phosphorus on the cell wall, disrupting the processes of respiration and replication and ultimately leading to cell death [72,73,74].

### 2.11. In Vitro Wound Scratch Assay

**Figure 14:**
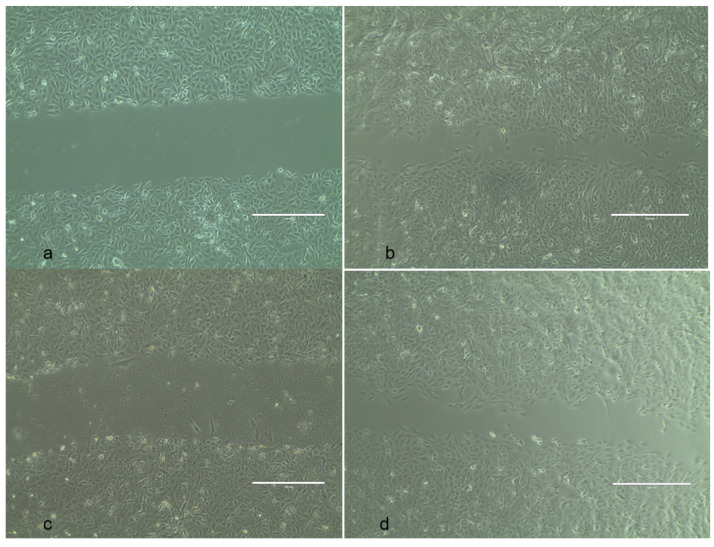
In vitro wound scratch assay. (**a**) Wounded cells (untreated), (**b**) control (treated with commercial drug), (**c**) treated with PVA, and (**d**) treated with AgNP-PVA nanofiber. The size measurement of the scale bar line is 500 μm (**a**–**d**).

The wound-healing efficacy of the mycosynthesized AgNPs and the commercial drug was observed under an inverted microscope, as shown in Figure 14, and the percentage of wound healing was calculated. The wound-healing efficacy of 20% was reported in 24 h with the mycosynthesized AgNPs, which is higher when compared to commercial drugs. This may be due to the presence of PGA-AgNPs, where PGA in this current study also works to improve the healing process [75]. The appearance of the repaired cells resembled that of unhealed cells. It was concluded, as a result, that AgNPs have a remarkably high healing capacity. According to previous findings, wound healing appears to be positively impacted by antibacterial and antioxidant activities [76]. Moreover, because they start the proliferative phase of repair, cell migration and proliferation are essential to the healing process [77].

## 3. Materials and Methods

### 3.1. Collection of Experimental Macrofungus

**Figure 15:**
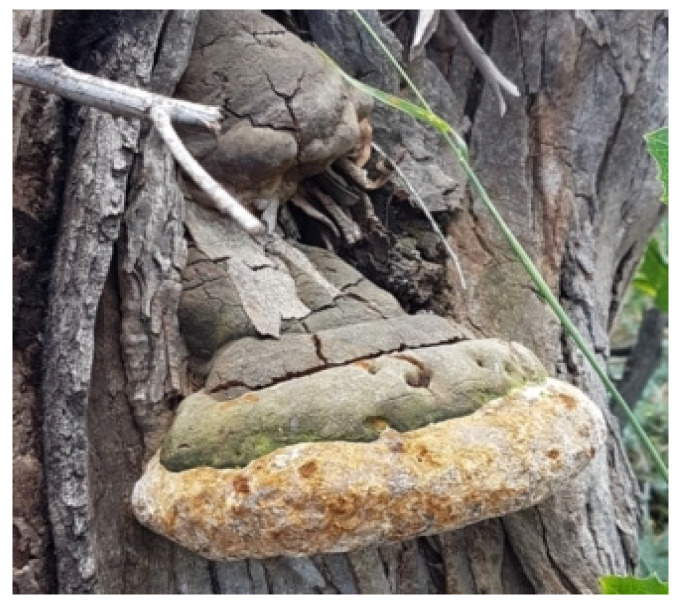
*Macrofungus sample Phellinus adamantinus* habitated with the trunk (stem) of an Angiosperm tree host.

Four different healthy fruiting bodies of the mushroom samples, namely *Schizophyllum commune* Fr., *Volvariella volvacea* (Bul.) Singer, *Phellinus adamantinus* (Berk.) Ryvarden, and *Inonatus porrectus* Murr., were collected in Kolli hills, Namakkal district, Tamil Nadu. The collected mushrooms were identified using the standard manuals (Figure 15).

### 3.2. Preparation of Aqueous Extract from Macrofungus

The fruiting bodies of the collected macrofungus (*Phellinus adamantinus*) were thoroughly washed under running tap water, followed by sterile distilled water, and then dried under shades. The dried samples were cut into several small pieces and powdered using a mortar and pestle. For each dried mushroom sample, 4 g was added to 50 mL of distilled water, then boiled at 55 °C for 15 min and cooled to reach room temperature. Aqueous extracts were filtered using Whatman No. 1 filter paper and refrigerated at 4 °C for further studies [44,58].

### 3.3. Mycosynthesis and Screening of AgNPs

In order to synthesize AgNPs, 1 mM AgNO_3_ was prepared. The fungal mushrooms of four different species (viz. *Schizophyllum commune* Fr., *Volvariella volvacea* (Bul.) Singer, *Phellinus adamantinus* (Berk.) Ryvarden, and *Inonatus porrectus* Murr.) were collected from the natural habitats at Kolli hills, Tamil Nadu, India and were used for extraction. The prepared mushroom extracts were mixed with distilled water in a ratio of 1:9 and treated with 20 μL of 1 mM AgNO_3_. For 24 h, these mixes were incubated at 37 °C in a dark static environment. The dark condition inhibits the incidence of photochemical reactions during the biosynthesis of nanoparticles. Following incubation, the mixture was compared to extracts devoid of AgNO_3_, which served as a control, and the color change from colorless to reddish brown was noted [52,78]. The produced AgNPs were measured using a UV-Vis spectrophotometer (U2900, Hitachi, Tokyo, Japan) between 300 and 700 nm in wavelength. The AgNPs synthesized from *Phellinus adamantinus* extract exhibited good stability among the four samples evaluated. Therefore, AgNPs produced by *Phellinus adamantinus* were screened further with different analyses [79].

### 3.4. Optimization of Mycosynthesized AgNPs

To achieve rapid and maximum synthesis of silver nanoparticles, different parameters (viz. pH, temperature, and concentrations of silver nitrate and fungal extract) were optimized.

#### 3.4.1. Effect of pH

An aqueous extract of *Phellinus adamantinus* (1 mL) was combined with 9 mL of 1 mM AgNO3 solution. Using hydrochloric acid and sodium hydroxide solutions, the mixture was brought to various pH values (3, 5, 7, 9, and 11) and kept at room temperature. After 24 h, the absorbances of the resulting mixes were measured using a UV-Vis spectrophotometer between 300 and 700 nm. The greater stability for pH 7 was noted, and the same was maintained in further studies [79].

#### 3.4.2. Effect of Temperature

First, 1 mL of aqueous extract of *Phellinus adamantinus* was added to 9 mL of 1 mM AgNO_3_ solution. The pH of the mixture was adjusted to 7 and incubated at various temperatures (4 °C, 22 °C, 37 °C, and 50 °C) for 24 h. The incubated mixtures were examined using a UV-Vis spectrophotometer between 300 nm and 700 nm. The synthesis of AgNPs was rapid and stable at 37 °C; hence, this study was carried out at 37 °C [80].

#### 3.4.3. Effect of Different Concentrations of Mushroom Extract

Different volumes of aqueous extract of *Phellinus adamantinus* (1 mL, 2 mL, 3 mL, 4 mL, and 5 mL) were added to 1 mM AgNO_3_ solution to make up 10 mL. The mixture was maintained at pH 7 and incubated at 37 °C. After 24 h of incubation, the synthesized AgNPs were observed using a UV-Vis spectrophotometer between the range of 300 nm to 700 nm. Among all five mixtures, 1 mL aqueous extracts were found to be stable and hence used for this study [65,81].

### 3.5. Characterization of Synthesized Silver Nanoparticles

#### 3.5.1. UV-Vis Spectroscopy

The synthesis of silver nanoparticles was observed using UV-Visible spectroscopy using a Motras scientific UV-Visible double-beam spectrophotometer. The formation of a reddish-brown color confirmed the synthesis of AgNPs. This gradual color change by a reduction process was evaluated and recorded using a UV-Visible spectrophotometer between 300 and 700 nm [82].

#### 3.5.2. TEM Analysis

TEM (transmission electron microscopy) (FEI Tecnai, Hillsboro, OR, USA) was performed to characterize the size and shape of the synthesized AgNPs at 100 and 200 scales [83]. TEM images were determined at an accelerating voltage of 300 kV. This study clearly exhibits the formation of polydisperse spherical and different magnitudes of crystalline AgNPs ranging from 20 to 50 nm in diameter. To prepare samples for analysis, 1 mg of isolated and dried mycosynthesized AgNPs was added to 1 mL of double-distilled water, then sonicated and spread on copper-coated carbon grids. TEM images were recorded.

#### 3.5.3. EDX Analysis

Elemental compositions of nanoparticles were determined by energy-dispersive X-ray spectroscopy (EDX analysis—(FEI Tecnai, Hillsboro, OR, USA)) [52]. The presence of AgNPs was confirmed by EDX spectra [84]. The mycosynthesized AgNPs were dried in a powdered form and estimated by using EDX analysis. This analysis revealed the purity of the synthesized AgNPs. The impurity of the sample can be determined when no peaks are obtained.

### 3.6. Antibacterial Activity

The well diffusion assay was used to assess the synthetic AgNPs’ antibacterial efficacy. As test organisms, four distinct human pathogenic bacteria were used, including the Gram-negative bacterium *Escherichia coli* and the Gram-positive bacteria *Bacillus subtilis* and *Staphylococcus aureus*. The nutrient agar plates were covered with a swab of the recently created pathogenic bacterial culture. With a sterile cork borer, four wells measuring 6 mm in diameter were created. The corresponding wells were loaded with 50 µL of AgNPs and mushroom extract and 20 mL of AgNO_3_ and streptomycin as the positive control. For 24 h, the loaded plates were incubated at 37 °C. The plates were incubated, and the area of inhibition that developed around the wells was measured in millimeters [85].

#### Minimum Inhibitory Concentration (MIC) of AgNPs

The broth microdilution method was performed in our study to evaluate the minimum inhibitory concentration (MIC) of synthesized silver nanoparticles, as stated in [86]. The broth microdilution method was done using 96-well microtiter plates. Fresh cultures of *Escherichia coli*, *Pseudomonas aeruginosa*, *Bacillus subtilis*, and *Staphylococcus aureus* were prepared using nutrient broth. Then, 100 µL of freshly prepared nutrient broth was loaded into each well and added with two-fold serial dilutions of synthesized silver nanoparticles with concentrations of 50, 25, 12.5, 6.25, and 3.125 µg/mL [56]. Then, 5 mL of inoculum was added to each well, and the microtiter plate was incubated at 37 °C for 24 h. This experiment was performed in duplicate. The growth of microbes inhibited by the lowest concentration of synthesized AgNPs was noted as the MIC.

### 3.7. In Vitro Cytotoxicity of Synthesized AgNPs

Vero (African green monkey kidney normal cell line) was obtained from the National Centre for Cell Sciences (NCCS), Pune, India. Cells were maintained in the logarithmic phase of growth in Dulbecco’s modified eagle medium (DMEM) supplemented with 10% (*v*/*v*) heat-inactivated fetal bovine serum (FBS), 100 U/mL penicillin, and 100 μg/mL streptomycin. They were maintained at 37 °C with 5% CO_2_ in a 95% air-humidified incubator. The cytotoxic effects of synthesized AgNPs were studied using a Vero cell line by 3-(4,5-dimethylthiazol-2-yl)-2,5 diphenyltetrazolium bromide (MTT) assay [87]. A 96-well microtiter plate was loaded with cells (1 × 10 ^6^ cells/mL) and incubated for 24 h at 37 °C in an incubator. After 24 h, the medium was removed, and the Vero cell lines were treated with different concentrations (10, 30, 50, 75, and 100 μg/mL) of synthesized AgNPs in duplicate. Cells without treatment with synthesized AgNPs were recorded as the control, and the microplate was incubated for 24 h with 5% CO_2_. After an incubation of 24 h, 20 μL of MTT solution was added to the cells and kept static for 4 h at 37 °C in a dark environment. Then, 100 μL of dimethyl sulphoxide (DMSO) was added, and the absorbance was recorded using a microplate reader. The cell viability was denoted in percentage (%) by

$$Cell\;viability\;(\%)=\frac{Absorbance\;of\;treated\;cells}{Absorbance\;of\;control\;cells}\times 100$$

### 3.8. Synthesis of Ag-PGA Nanoparticles and Preparation of (Ag-PGA)-PVA Spinnable Solution

A 1% (*w*/*v*) solution was prepared by diluting polygalacturonic acid (PGA) with double-distilled water. Then, 1 mM NaOH was added to the mixture, and a magnetic stirrer was employed for complete dissolution. After 30 min, 400 μL of 0.45 mM AgNO_3_ was added to the mixture. A yellow solution was obtained after 1 h under continuous stirring [46]. Then, 1% Ag-PGA dry powder was added to dissolve 8% (*w*/*v*) poly vinyl alcohol (PVA) at 30 °C and subjected to continuous stirring until a homogenous solution was obtained [88].

### 3.9. Synthesis of Ag-PGA Nanoparticles and Preparation of (Ag-PGA)-PVA Spinnable Solution

A 1% (*w*/*v*) solution was prepared by diluting polygalacturonic acid (PGA) with double-distilled water. Then, 1 mM NaOH was added to the mixture and a magnetic stirrer was employed for complete dissolution. After 30 min, 400 μL of 0.45 mM AgNO_3_ was added to the mixture. A yellow solution was obtained after 1 h under continuous stirring [46]. Then, 1% Ag-PGA dry powder was added to dissolve 8% (*w*/*v*) PVA at 30 °C and subjected to continuous stirring until a homogenous solution was obtained [88].

### 3.10. Electrospinning

The spinnable solution was put into a glass syringe fitted with a metal capillary needle (0.5 mm inner diameter). An alligator chip held this solution to the needle. The ground plate (covered with aluminum foil) and collector make up the electrospinner, together with the syringe pump. Working solutions are inserted using a syringe pump with a flow rate of 1.3 milliliters per hour, and a high voltage of around 11 kV was utilized for electrospinning at room temperature. The aluminum foil’s nanofibers were cured at ambient temperature [69].

### 3.11. In Vitro Wound Scratch Assay

The effectiveness of the synthesized silver nanoparticles in healing wounds was assessed using a wound scratch assay. Under ideal conditions, the healthy Vero cells were cultured in a 12-well plate until they reached 90% confluence. A sterile pointed needle was used to simulate a wound and create a scratch at the center of the well to cause damage to the cell monolayer. To get rid of cell debris, the cells were cleaned using a PBS buffer. The incision was treated with AgNP-coated nanofiber, PVA nanofiber, and 50 μg/mL of cipladine (positive control). The incision that was left untreated was regarded as the negative control and was incubated at 37 °C for 48 h with 5% CO_2_. Under an inverted microscope, the wound closure was inspected.

The digital pictures were captured at time 0 (T0), 24 h (T1), and 48 h (T2). With the use of imageJ 1.8.0 processing software, the wound closure was calculated by the difference between the breadth of the wound at the beginning and the end of the healing phase [44,89]. The distance between the wounded widths at T0 and T1/T2 was used to quantify the scratch’s closure. The formula used to obtain the scratch closure rate (SCR) is as follows:

SCR = ((T0 − T1/T2)/T0) × 100

where T0 is the scratch area at time 0, and T1/T2 is the scratch area at 24 h and 48 h.

## 4. Conclusions

Using an aqueous extract of *Phellinus adamantinus*, macrofungus (mushroom)-mediated AgNPs were successfully synthesized. A report on the biosynthesis of nanosilver with this mushroom species was recorded for the first time in science. UV-Vis spectroscopy was used to analyze the synthesized AgNPs, and the results revealed good stability at 420 nm over 40 days. AgNP morphology and particle size were described via TEM. The average size of the spherically shaped particles ranged from 40 to 50 nm, which is smaller in comparison with earlier reports. Pure silver nanoparticles are present since an absorption peak at 3 keV is shown by EDX. By adjusting the silver nitrate and mushroom extract concentrations, the synthesized AgNPs were adjusted for a range of pH values. At 37 °C and pH 7, the AgNPs remained stable. It was discovered that the ideal concentrations of mushroom extract and silver nitrate were 1 mM and 1 mL, respectively. The bioactive principle of fungal extract has a major role in causing doping and reduction function in the synthesis of durable and functionalized AgNPs. The antibacterial assay against test species like *Bacillus subtilis* (4 mm), *Escherichia coli* (3 mm), *Pseudomonas aeruginosa* (4 mm), and *Staphylococcus aureus* (11 mm) showed that the improved AgNPs demonstrated possible bactericidal action. After determining the minimum inhibitory concentration (MIC) for each of the four test species, which included bacteria, it was discovered to be 3.125 µg/mL. After analyzing the in vitro cytotoxicity using the MTT test, the optimized AgNPs’ IC50 was determined to be 100 µg/mL. It was reported that the optimized AgNP-coated nanofiber was effective at healing wounds. This study will establish an innovative, ecofriendly route to the green synthesis of nanosilver with unique chemical and physical properties, which could lead to the development of novel microbicidal and wound-healing therapeutic agents.

## Figures and Tables

**Table 1:** The diameter of the antibacterial inhibition zone of AgNP/PGA/PVA composite nanofibers against *E. coli* and *S. aureus.*

Samples	Diameter of Antibacterial Inhibition (mm)	
	*E. coli*	*S. aureus*
0% AgNPs	0	0
1% Ag-PGA with 8% PVA	8.1	2.2

## Data Availability

Data are contained within the article.
